# Hospital costs of maternal near miss: a micro-costing analysis

**DOI:** 10.31744/einstein_journal/2025GS0933

**Published:** 2025-07-30

**Authors:** Moara Maria Silva Cardozo, Suely Arruda Vidal, Arthur Lima Xavier de Azevedo

**Affiliations:** 1 Instituto de Medicina Integral Prof. Fernando Figueira Postgraduate Program in Integral Health Recife PE Brazil Postgraduate Program in Integral Health, Instituto de Medicina Integral Prof. Fernando Figueira, Recife, PE, Brazil.; 2 Faculdade Pernambucana de Saúde Recife PE Brazil Faculdade Pernambucana de Saúde, Recife, PE, Brazil.

**Keywords:** Costs and cost analysis, Cost of illness, Pregnancy complications, Near miss, healthcare, Maternal health

## Abstract

**Objective:**

This study aimed to estimate the hospital costs of maternal near miss cases from both the institutional and Brazilian Public Health System perspectives in a reference maternitiy hospital.

**Methods:**

This cost-of-illness study used primary data collected from 128 patients admitted to the obstetric intensive care unit of a reference hospital in 2019 who met the maternal near-miss criteria. Two costing methods were used: micro-costing assessment through reviewing medical records and micro-costing assessment derived from reimbursement data of the Brazilian Public Health System. The resources used were assigned costs in reais (R$) that were converted into US dollars (US$) based on 2019 values.

**Results:**

The average cost of a hospital stay for patients treated for maternal near miss was US$ 1,427.70 per medical record review and US$ 973.00 per Brazilian Public Health System reimbursement. The costs estimated by reimbursement were significantly lower than those estimated by reviewing medical records (p<0.0001). Considering the estimated incidence of maternal near miss among women treated in the Brazilian Public Health System, the projected national economic burden was US$ 86,055,130.50 in 2019.

**Conclusion:**

Through using a micro-costing methodology, this study established that, for the Brazilian Public Health System, hospital admissions due to maternal near misses had resulted in high costs, although this condition is considered to involve a large number of preventable cases. The cost estimates derived from the institution's medical records were significantly higher than the estimated reimbursement costs.

## In Brief

This micro-costing study assessed hospital admissions for maternal near miss in a high-complexity maternity referral center in Brazil. The findings revealed an average cost of US$ 1,427.70 per admission, with personnel costs being the main driver. The estimated national economic burden reached US$ 86 million in 2019. As most cases are preventable, this underscores the systemic gaps in maternal healthcare delivery and reinforces the need for targeted policy interventions to improve quality and prevent avoidable complications.

## Highlights

■ Micro-costing showed high per-admission costs (US$ 1,427.70) for maternal near miss.
■ Reimbursement values (US$ 973) underestimate true hospital costs.
■ Brazil's economic burden estimated at US$ 86 million (2019).
■ Most maternal near miss events stem from preventable causes.

## Graphical abstract

**Figure:**
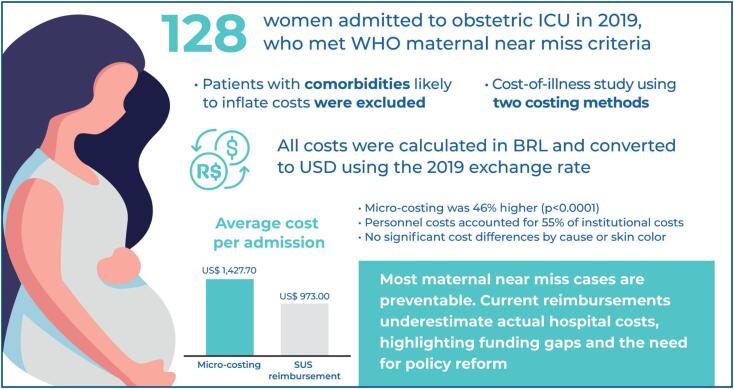

## INTRODUCTION

Women who almost die but who survive a serious maternal complication that occurs during pregnancy, childbirth, or the postpartum period have experienced a condition defined by the World Health Organization (WHO) as "maternal near miss" (MNM).^(1)^ Investigation of this condition is essential because although the maternal mortality ratio is widely recognized, it fails to capture the full spectrum and severity of maternal morbidity.^(2,3)^

In Brazil, the MNM ratio varies from 2.4/1000 live births (LB) to 188.4/1000 LB depending on the criteria applied and the epidemiological scenarios considered.^(4)^ Inequalities in maternal health quality also appear to be associated with MNM events. In Brazil, values are three times higher in regions with the lowest human development index and non-white women develop more severe conditions more frequently, while and a greater number of prenatal consultations are recognized as a protective factor for MNM.^(5)^

Maternal death is an event that deserves attention, not only because of the repercussions within the family, but also because most are considered preventable.^(6,7)^ It has been reported that 95% of maternal deaths worldwide can be avoided, which demonstrates the importance of identifying deficiencies in women's care.^(8)^ Furthermore, if spending on MNM could be reduced, more financial support would be available to address other clinical conditions or in other sectors of society, which in health economics is defined as opportunity cost.

Given the importance of information derived from health evaluation studies, estimating the expenses involved with this condition and understanding the magnitude of the economic burden associated with MNM is essential in relation to determining effective strategies to strengthen obstetric care.

## OBJECTIVE

This study aimed to estimate and compare the hospital costs of patients admitted with maternal near miss to a reference maternity hospital in northeastern Brazil from the perspective of the paying institution and of the Brazilian Public Health System.

## METHODS

### Study design, location and period

This cost-of-illness study was undertaken with consideration of the perspective of the paying institution and of the Brazilian Public Health System (SUS - *Sistema* Único *de Saúde*). The study was conducted at the *Instituto de Medicina Integral Prof. Fernando Figueira* (IMIP), a quaternary reference hospital for high-risk maternal care and childcare, between January and November 2023. The IMIP provides care, on average, in relation to 5,000 births per year, with an obstetric intensive care unit (ICU) comprising 10 beds that is exclusively for pregnant and postpartum women at high risk. Approximately 70 women are admitted to the obstetric ICU per month, of whom 15%-20% meet WHO criteria for MNM.

### Population and sample

The study population comprised women admitted to the obstetric ICU who met WHO criteria for MNM. Patients with comorbidities likely to substantially inflate costs unrelated to MNM were excluded (those with malignant neoplasms, chronic kidney disease requiring renal replacement therapy, chronic heart disease, and acquired immunodeficiency syndrome). The final sample comprised 128 patients.

### Cost components

Direct medical costs were collected in this study using the micro-costing method. Direct medical costs were defined as those resulting from the following health interventions: hospital services (hospitalization in the ward/ICU), professional services, medications, and radiological and laboratory tests.

The costs were calculated in reais (R$) and converted into US dollars (US$) using the 2019 average exchange rate (US$1= R$4.1831), according to the Central Bank of Brazil.

### Costing methods considered

Two costing methods were used: a micro-costing/bottom-up method applied through a review of medical records, and a micro-costing/top-down method applied through using reimbursement data obtained from the SUS.

### Costing derived from the medical record review data

To determine direct medical costs using the micro-costing/bottom-up method, patient medical records data were used, from which information was obtained on medications administered, laboratory tests, imaging tests, and hospitalization in a ward or care unit involving intensive therapy.

The elements that constituted care costs were quantified; all medications administered were counted in terms of frequency of administration and estimates derived in terms of the unit value of each item. The cost of the medications was obtained from average charges information as applied by the Health Prices Bank. Laboratory tests were performed by an external laboratory at the same cost as that on the SUS fixed-price list.

To estimate personnel costs during hospitalization, we obtained data from the hospital's financial department on the average monthly salaries of obstetric physicians, nurses, and nursing technicians at IMIP, without identifying individual professionals. A standardized labor cost adjustment of 30.19% was applied to account for employment-related obligations. The resulting amount was then proportionally allocated to each patient, based on the average number of monthly hospitalizations in the relevant units.

### Costing derived from the reimbursement data

Hospital admission authorization (AIH - *Autorização de Internação Hospitalar*) for each patient was obtained from the Medical Accounts Department. The amounts reimbursed by the SUS for each patient for hospital services, daily companion fees, professional services, and physiotherapy were identified and analyzed to estimate the hospitalization costs for each patient.

### Data analysis

Demographic data are described as mean and standard deviation (SD). For each method, the average cost of treating patients for MNM was calculated. To test the difference in costs between those determined for the institution and for reimbursement from the SUS, a paired *t*-test was used in relation to OpenEpi version 3.01 software.

### Ethical aspects

This study was approved by the Ethics Committee of IMIP (CAAE: 38626920.8.0000.5201; #4.420.542). As it was a retrospective observational study using data from medical records and institutional databases, a waiver of informed consent (*Termo de Consentimento Livre e Esclarecido* - TCLE) was requested and granted.

## RESULTS

In total, 128 patients were included in the cost analysis. Their average age was 28.9 years (range: 13-45 years). Notably, 62.2% of the patients were categorized as black or of mixed race. The majority (56.7%) had between 7 and 12 years of education. Regarding the type of delivery, 88.2% underwent cesarean section. Regarding MNM events by cause, 61.7% had complications related to hypertension, 15.6% had hemorrhage, 11.7% had infection, and 11% had other causes. The clinical and epidemiological characteristics of the patients included in the study are presented in table 1.

**Table 1:** Clinical-epidemiological characteristics of patients hospitalized for maternal near miss

Characteristics		%
Age (years)		
	<20	14
	20-40	82
	>40	4
	Average (±SD)	28.9 (±8.4)
Skin color		
	Black/brown	62.2
	White	37.8
Marital status		
	Married/stable union	77.3
	Single/divorced	22.7
Profession		
	Housewife	52.6
	Public employment	5.3
	Private employment	15.8
	Farmer	2.6
	Domestic	5.3
	Student	10.5
	Unemployed	7.9
Years of study		
	0-6	24.3
	7-12	56.7
	>12	19
Cause of MNM		
	Bleeding	15.6
	Infection	11.7
	Hypertension	61.7
	Other	11
Length of stay (days)		
	<7	14.1
	7-14	47.6
	>14	38.3
	Average (±SD)	14.1 (±10.9)
Type of birth		
	Vaginal	11.8
	Cesarean section	88.2

SD: standard deviation; MNM: maternal near miss.

Some of the main health resources and the percentages of the sample that used them are shown in table 2. Most patients were prescribed with analgesics, dimethicone, or anticoagulants. Among the imaging tests, ultrasonography was the most common (52.3%). A small proportion of the patients required hemodialysis (3.9%).

**Table 2:** Type of health resource and percentage of the sample that used it among patients hospitalized for maternal near miss

Health resources		n (%)
Medications		
	Analgesic	119 (92.9)
	Dimethicone	92 (71.9)
	Anticoagulant	71 (55.5)
	Antibiotic	50 (39.1)
	Proton pump inhibitor	49 (38.3)
Imaging exams		
	Ultrasound	67 (52.3)
	Tomography	17 (13.3)
	MRI	07 (5.5)
Other Procedures		
	Blood transfusion	45 (35.1)
	Surgical procedures	27 (21.1)
	Hemodialysis	05 (3.9)

MRI: magnetic resonance imaging.

### Maternal near miss costs using different costing methods

The cost of hospital admission for patients treated for MNM varied depending on the method used, resulting in an average cost of US$1,427.70 per medical record review and US$973.00 per SUS reimbursement. These latter values were significantly lower than those estimated from the medical record review (p<0.0001).

As shown in table 3, from the institution's perspective, personnel expenses were responsible for the largest portion of the total costs, representing 54.95%.

**Table 3:** Distribution of expenses in relation to maternal near miss from the perspective of the institution

Item	Cost (US$)	%
Personnel expenses	100,422.64	54.95
Fixed costs	26,637.33	14.57
Childbirth	20,530.07	11.23
Medicines	10,902.89	5.96
Consumables	10,575.73	5.78
Laboratory tests	5,862.67	3.20
Surgical procedures	5,836.77	2.94
Imaging exams	1,238.88	0.70
Blood transfusion	262.74	0.14
Total	182,746.67	100

As shown in table 4, from the perspective of the SUS, the highest expenses when grouped were due to patients with complications secondary to hypertensive emergencies (US$ 74,699.47). There were no statistically significant differences in the means of the groups according to cause (bleeding, hypertensive emergency, and infection) (p=0.67). We also found no differences when comparing the hospitalization costs of black/brown patients in relation to white patients (p=0.88).

**Table 4:** Total expenses and per capita costs due to the most frequent maternal near miss causes

Cause of MNM	Total expenses (US$)	Average/patient (US$)
Hypertension	74,699.47	995.99
Bleeding	10,245.29	539.22
Infection	18,857.36	1257.15

MNM: maternal near miss.

Based on data obtained from a study that estimated the incidence of MNM among women treated within the SUS in 2019, we calculated an economic impact of US$86,055,130.50 for the system.

## DISCUSSION

In the present study, the average cost for the paying institution was US$ 1427.70 and US$ 973.00 for the SUS in relation to treating MNM per patient. When considering this average cost in terms of the estimated incidence of MNM in Brazil in 2019, the annual cost of treating this condition was US$86,055,130.50.

Few studies in the literature have estimated the costs related to MNM.^(9-12)^ A study conducted in Kenya identified costs from the patient's perspective, directly through interviews, and found that the cost was 17 times higher for patients who had complications with MNM than for those who had an uncomplicated vaginal birth.^(12)^

An American study created a model capable of estimating the costs of MNM cases from the beginning of pregnancy up to five years postpartum in terms of evaluating maternal and child outcomes, with medical costs of US$18,723.70 and US$13,576.80, respectively, during this period.^(13)^ Most of the maternal costs were due to lost productivity, cesarean section, and increased peripartum hospitalization time. Monitoring over a longer time horizon makes it possible to identify expenses due to later consequences from MNM for women and children in terms of physical and mental health, including consequences for subsequent pregnancies.

Data from the Brazilian Obstetric Observatory released in 2022 indicate that nine out of ten maternal deaths are preventable with timely access to evidence-based maternal care, including contraception.^(14)^ As a large proportion of MNM cases are caused by the same causes of death, these cases are also potentially preventable. Spending on this condition should be interpreted as a possibility of savings for the health system and society, which could lead to better allocation of resources to other treatments or even other sectors.

The results found in the studied sample in relation to color/race and MNM due to hypertensive complications are compatible with information released by the Ministry of Health, which highlights that maternal mortality in Brazil disproportionately affects black women.^(15,16)^ Similarly, maternal death from hypertension was reported to have increased by 5% among black women between 2010 and 2020, while there was a drop in other groups.^(17)^ Such findings reinforce the importance of addressing maternal health as an indicator of inequity in population health.

Estimates of the cost of illness are necessary to improve precision in cost-effectiveness studies.^(18,19)^ Few national studies have compared SUS reimbursement costs with other costing methodologies, but there is a common belief that reimbursement underestimates the real cost of hospitalizations.^(20,21)^ Our study revealed a significant difference between the two types of costing assessed.

The SUS procedure table applies an average hospital stay of three days for admissions related to childbirth in high-risk pregnancies, which is lower than the average length of stay found in our sample (14.1 days). Other research on different medical conditions has also highlighted possible underestimation of what typically occurs in hospitals.^(22)^

Although the cost per medical record (micro-cost) is considered the gold standard owing to its greater accuracy, it requires more time and resources to assess effectively.^(23)^ However, a mixed approach could be used to prioritize micro-costing for the components that have the greatest impact on the total cost.^(24)^

This study has some limitations. First, due to its retrospective design, direct nonmedical costs from the patient's perspective—as well as indirect costs—were not included. Second, extrapolating cost data from a single institution to the national level provides only approximate estimates. Future studies should address these gaps to enhance the robustness and generalizability of the findings.

The findings of this study confirm that women who have complications due to MNM generate high costs from the perspective of hospital admission (for the institution and for the SUS). Considering that a large number of such complications are avoidable, an accurate estimate of the economic burden attributable to MNM can facilitate a deeper awareness of its impact on society and help prompt public policies that prioritize more extensive maternal care.

## CONCLUSION

Using a micro-costing approach, this study found that hospital admissions for maternal near miss in Brazil incurred high costs in 2019—a concerning finding given the largely preventable nature of these cases. Cost estimates based on institutional data were substantially higher than those reimbursed by the Brazilian Public Health System, suggesting a potential underestimation of actual expenditures within the national system.
